# The Association between Sarcopenic Obesity and Depressive Symptoms in Older Japanese Adults

**DOI:** 10.1371/journal.pone.0162898

**Published:** 2016-09-14

**Authors:** Shinya Ishii, Chang Chang, Tomoki Tanaka, Aki Kuroda, Tetsuo Tsuji, Masahiro Akishita, Katsuya Iijima

**Affiliations:** 1 Department of Geriatric Medicine, Graduate School of Medicine, The University of Tokyo, Tokyo, Japan; 2 Institute of Gerontology, The University of Tokyo, Tokyo, Japan; Ehime University Graduate School of Medicine, JAPAN

## Abstract

The effects of sarcopenic obesity, the co-existence of sarcopenia and obesity, on mood disorders have not been studies extensively. Our objective was to examine the association of depressive symptoms with sarcopenia and obesity status in older Japanese adults. We analyzed data from 1731 functionally-independent, community-dwelling Japanese adults aged 65 years or older (875 men, 856 women) randomly selected from the resident register of Kashiwa city, Chiba, Japan in 2012. Sarcopenia was defined based on appendicular skeletal muscle mass, grip strength and usual gait speed. Obesity was defined as the highest sex-specific quintile of the percentage body fat. Depressive symptoms were defined as a Geriatric Depression Scale 15-item score ≥ 6. Multiple logistic regression was employed to examine the association of depressive symptoms with four groups defined by the presence/absence of sarcopenia and obesity. The prevalence of depressive symptoms was 10.1% and the proportions of sarcopenia/obesity, sarcopenia/non-obesity, non-sarcopenia/obesity, non-sarcopenia/non-obesity were 3.7%, 13.6%, 16.9% and 65.8%, respectively. After adjustment for potential confounders, sarcopenia/obesity was positively associated with depressive symptoms compared with non-sarcopenia/non-obesity, whereas either sarcopenia or obesity alone was not associated with depressive symptoms. The association was particularly pronounced in those aged 65 to 74 years in age-stratified analysis. We conclude that our findings suggest a synergistic impact exerted by sarcopenic obesity on the risk of depressive symptoms, particularly in those aged 65 to 74 years.

## Introduction

Obesity and depression are considered major threats to public health worldwide. Obesity has more than doubled since 1980 and is considered a major risk factor for various diseases including cardiovascular diseases, diabetes, osteoarthritis and cancers[1]. Depression is prevalent and a major risk factor for suicide, and is associated with considerable morbidity and mortality [2].

The association between these two major medical conditions has attracted much research interest. However, epidemiological studies to date have generated conflicting results. Some studies showed a positive association between depression and obesity [3], but others showed an inverse association [4] or U-shaped association [5]. Some studies even failed to show a significant association [6]. This inconsistency may have resulted from differences in study design, subject population, depression measurement, statistical analysis or covariates used. Another possible explanation could be related to the limited discrimination ability of body mass index (BMI) (i.e., ability to correctly classify obesity), which was used in most studies to ascertain obesity.

Obesity is abnormal or excessive fat accumulation that may impair health [1]. While BMI is the most useful measure of obesity at the population level, it does not differentiate muscle mass from fat in individuals. Individuals with low BMI may still have as much fat as those with high BMI, and higher BMI may mean greater muscle mass in some individuals. Use of BMI, an imperfect measure of adiposity, is actually considered as one of the explanations for the obesity paradox, the unexpected phenomenon that overweight or mild obesity is associated with decreased mortality compared with normal weight [7,8]. Indeed, recent large-scale prospective cohort study demonstrated that both low BMI and high body fat percentage are independently associated with increased mortality, claiming the importance of using direct measures of adiposity [9].

Use of BMI is particularly problematic when used to identify obesity in the elderly. People accumulate fat while losing lean body mass as they age. Hence, the discrimination ability of a certain BMI cut-off point with respect to body adiposity decreases with age [10]. In addition, body composition is highly heterogeneous in the elderly [11], which may weaken the association between BMI and body adiposity.

The loss of muscle mass and physical function with age needs to be accounted for as well in studying factors associated with depression in the elderly [6]. Lower muscle mass appears to be associated with increased depressive symptoms [12]. Several prospective cohort studies demonstrated a role of decreased physical performance or physical activity in the development of depression in the elderly. Lower physical performance measured as gait speed [13,14] or grip strength [15] was associated with the development of depression. Therefore, it is reasonable to hypothesize that sarcopenia, a syndrome characterized by progressive, generalized loss of skeletal muscle mass and strength with a risk of adverse outcomes [16], may be associated with depression.

In the present study, we employed percentage body fat to determine obesity, and examined the association of depressive symptoms with sarcopenia and obesity status in community-dwelling Japanese elderly. We hypothesized that both obesity and sarcopenia are associated with depressive symptoms, and that sarcopenic obesity, the co-existence of sarcopenia and obesity, is associated with higher risk of depressive symptoms compared with either alone, considering that sarcopenic obesity may carry cumulative risk derived from each of these two individual body composition phenotypes.

## Materials and Methods

### Participants

We analyzed data from the participants of the Kashiwa study, which is designed to characterize the biological, psychosocial and functional changes associated with aging in community-dwelling older adults. The sampling procedure was described in detail elsewhere [17]. Briefly, the inclusion criteria for study entry were age 65 years or older and functional independence (i.e., not requiring nursing care provided by long-term care insurance). The participants were randomly selected from the resident register of Kashiwa city, Chiba, Japan. A total of 2044 older adults (1013 men, 1031 women) were enrolled and underwent baseline survey in 2012. Participants with missing values of any variables were excluded from the study, leaving the analytic sample of 1731 older adults (875 men, 856 women).

The study was approved by the ethics committee of the Graduate School of Medicine, The University of Tokyo. All participants provided written informed consent.

### Measures

#### Independent variables

Sarcopenia was defined based on the presence of low muscle mass plus the presence of either low muscle strength or low physical performance [16].

Muscle mass was measured by bioimpedance analysis using an Inbody 430 machine (Biospace, Seoul, Korea). Appendicular skeletal muscle mass (ASM) was derived as the sum of the muscle mass of the four limbs. ASM was then normalized by height in meters squared to yield skeletal muscle mass index (SMI) (kg/m^2^). SMI values lower than two standard deviations below the mean values of young male and female reference groups were classified as low muscle mass (SMI < 7.0 kg/m^2^ in men, < 5.8 kg/m^2^ in women) [18].

Muscle strength was assessed by hand grip strength, which was measured using a digital grip strength dynamometer (Takei Scientific Instruments, Niigata, Japan). Measurement was performed twice using the dominant hand, and the higher of two trials (in kilograms) was used for the present analysis. Hand grip strength values in the lowest quintile were classified as low muscle strength (cutoff values: 30 kg for men, 20 kg for women).

Physical performance was assessed by usual gait speed. Participants were instructed to walk over an 11-meter straight course at their usual speed. Usual gait speed was derived from 5 meters divided by the time in seconds spent in the middle 5 meters (from the 3-meter line to the 8-meter line). Good reproducibility of this measurement has been reported [19]. Usual gait speed values in the lowest quintile were classified as low physical performance (cutoff value: 1.26 m/s for each sex).

Participants were classified as obese if their percentage body fat measured by BIA was in the highest quintile (cutoff values: 29.7% for men, 37.2% for women).

Based on the presence/absence of sarcopenia and obesity, participants were categorized into four groups: sarcopenia/obesity, non-sarcopenia/obesity, sarcopenia/non-obesity, and non-sarcopenia/non-obesity.

#### Outcome

Depressive symptoms were assessed using the self-reported 15-item Geriatric Depression Scale (GDS). We defined depressive symptoms as a score of 6 or more and severe depressive symptoms as a score of 10 or more [20]. The GDS Japanese version has been validated for use in Japanese older adults with good psychometric properties, and a cut-off score of 6 or more had the highest discrimination ability, with sensitivity of 97.3% and specificity of 95.9% [21].

#### Covariates

Demographic information, medical history of doctor-diagnosed chronic conditions, use of medication, and living conditions were obtained using a standardized self-reported questionnaire. Participants were asked to rate their amount of food intake on the five-point Likert scale (“very large”, “large”, “normal”, “small”, or “very small”). Physical activity was assessed using the Global Physical Activity Questionnaire, and metabolic equivalents (METs)-minute per week was computed [22]. Chronic comorbidity burden was quantified as the number of self-reported chronic conditions including diabetes mellitus, hypertension, stroke, heart disease (excluding hypertension), dyslipidemia, chronic kidney disease, osteoporosis and cancer. Height and weight were measured with the participants wearing light clothing and no shoes, using a fixed stadiometer and a digital scale, and used to compute BMI.

Sleep quality was evaluated using the Pittsburgh Sleep Quality Index, of which Japanese version has been validated [23]. The total score ranges from 0 to 21, with a lower score indicating better sleep, and participants with a score higher than 5 are considered poor sleepers.

Social support provided by family/friends was assessed using the 6-item Lubben Social Network Scale. This scale asks about the number of family members/friends with whom one has social contact and social support, and is widely used as a validated screening tool for social isolation among community dwelling older adults [24]. The scale ranges from 0 to 30, with a higher score indicating a strong social support network, and participants with a score less than 12 are considered socially isolated.

Social connections beyond the social support provided by individual relationships with family and friends were assessed using the Social Cohesion Scale [25]. The Social Cohesion Scale asks five questions regarding the strength of neighborhood ties, such as whether people in the neighborhood can be trusted and whether they get along with each other. Each question was answered with a 5-point Likert scale, which was added to generate a total score ranging from 5 to 25, with a lower score representing greater social cohesion.

### Statistical Analysis

Differences in participant characteristics among the four groups defined by sarcopenia and obesity status were examined using ANOVA test (or Kruskal-Wallis test) for continuous variables and chi-square test for categorical variables. The p-values were adjusted for multiple testing using the Hochberg procedure.[26] To evaluate the association with depressive symptoms, odds ratios (ORs) with 95% confidence intervals (CIs) for depressive symptoms were obtained by conducting multiple logistic regression analysis while controlling for covariates.

Our preliminary analysis indicated that there was no evidence of effect modification by sex on the covariates-adjusted associations of depressive symptoms with sarcopenia and obesity status (Wald p = 0.72–0.78). Therefore, data for men and women were pooled and analyzed together. As the next step of the preliminary analysis, we tested the applicability of BMI as a measure of obesity in the context of researching factors associated with depressive symptoms. We conducted covariates-adjusted multiple logistic regression with BMI as an independent variable, in the form of a linear term, a quadratic term or a binary variable with a cutoff of 25, with or without sarcopenia, in addition to interaction terms with sarcopenia when sarcopenia was in the model, but BMI was not significantly associated with depressive symptoms in any model (data not shown).

In the main analysis, we conducted multiple logistic regression analysis with obesity ascertained using percentage body fat. Covariates were selected a priori based on their association with depression, sarcopenia or obesity and added to the model in a sequential manner. The model was initially adjusted for age and sex (model 1). We first added behavior variables including food intake (five-level categorical), physical activity and sleep quality (poor sleeper vs. good sleeper) (model 2). We further adjusted for social factors including living condition (living alone or not), education level (three-level categorical, “below high school”, “high school graduate”, “college or higher”), social isolation and Social Cohesion Scale (model 3). In the final model, medical variables including use of antidepressants, use of statin and chronic comorbidity burden were added to the model (model 4).

Several recent studies have suggested that age modifies the effects of cardiovascular risk factors [7,27–29]. Cardiovascular risk factors do not seem to exert adverse effects on the very old or frail individuals as they do on younger individuals. Analysis with an interaction term between age and sarcopenia and obesity status verified the presence of effect modification by age (Wald p < 0.001 in age- and sex-adjusted model, p = 0.02 in the fully-adjusted model). We then further divided participants into young-old (65–74 years) and old-old (≥ 75 years) as per previous studies [29] and conducted age-stratified analysis.

We defined depressive symptoms as GDS score of 6 or higher, but this definition may misclassify persons as being depressed when they are not, as a result of temporary stress or symptoms due to physical conditions. Therefore, we repeated all analyses with severe depressive symptoms as a dependent variable to test whether adoption of a higher cutoff point for GDS may alter the association of depressive symptoms with sarcopenia and obesity status.

Finally, because the effects of comorbid conditions on depressive symptoms may vary, we chose four medical conditions that are strongly associated with depressive symptoms, namely diabetes mellitus, stroke, heart disease, and cancer, and added the presence or absence of these medical conditions separately as a covariate.

All analyses were conducted using SAS version 9.3 (SAS Institute Inc., Cary, NC) and R statistical software version 2.15.2 (R Foundation, Vienna, Austria). Two-sided p < 0.05 was considered statistically significant.

## Results

### Participant characteristics

The prevalence of depressive symptoms was 10.1% (9.6% in men and 10.5% in women). The proportions of sarcopenia/obesity, sarcopenia/non-obesity, non-sarcopenia/obesity, and non-sarcopenia/non-obesity were 3.7%, 13.6%, 16.9% and 65.8%, respectively. The characteristics of the study participants by sarcopenia and obesity status are shown in Table 1. Those with sarcopenia/obesity tended to be older, less physically active, less educated, and had greater chronic comorbidity burden and poor sleep compared to those without sarcopenia or obesity.

**Table 1:** Participant characteristics by sarcopenia and obesity status in the Kashiwa study.

	Sarcopenia/Obesity	Sarcopenia/Non-obesity	Non-sarcopenia/Obesity	Non-sarcopenia/Non-obesity	p
	N = 64	N = 236	N = 292	N = 1139	
Age	77.1 (5.2)	76.9 (6.0)	72.9 (5.4)	71.8 (4.8)	<.001
Male	50%	36.9%	51.0%	53.3%	<.001
Physical activity	1980 (870, 3660)	2020 (930, 4000)	2570 (1360, 4970)	3120 (1680, 5400)	<.001
Education level					0.003
Below high school	25.0%	19.5%	13.4%	11.7%	
High school	43.8%	52.1%	50.7%	47.7%	
College or higher	31.3%	28.4%	36.0%	40.7%	
Living alone	12.5%	11.9%	13.7%	9.3%	0.65
Socially isolated	28.1%	22.5%	18.5%	19.8%	0.71
Social Cohesion Scale	13.7 (2.2)	13.8 (2.1)	14.0 (1.8)	14.1 (2.0)	0.65
Chronic comorbidity burden	1.9 (1.2)	1.6 (1.3)	1.7 (1.1)	1.3 (1.1)	<.001
Comorbidities					
Stroke	7.8%	7.6%	6.5%	5.1%	0.71
Diabetes	20.3%	10.6%	15.4%	10.2%	0.07
Heart disease	18.8%	24.2%	20.6%	14.5%	0.008
Cancer	17.2%	16.1%	15.1%	14.6%	0.89
Antidepressant use	1.6%	0.9%	1.7%	0.6%	0.71
Statin use	31.3%	22.9%	31.9%	21.3%	0.008
Poor sleeper	39.1%	41.5%	28.8%	25.9%	<.001
Food intake					<.001
Very large	1.6%	1.7%	2.7%	2.9%	
Large	10.9%	7.2%	20.6%	14.6%	
Normal	65.6%	61.4%	67.5%	71.5%	
Small	21.9%	25.4%	8.9%	9.9%	
Very small	0%	4.2%	0.3%	1.1%	

Mean and standard deviation are shown for continuous variables, except for physical activity whose distribution was highly skewed and therefore median and interquartile range are shown for physical activity. Proportions as percent are shown for categorical variables. Percentages may not add up to 100 because of rounding.

p-values for group differences were calculated using ANOVA test for continuous variables (or Kruskal-Wallis test for physical activity) and chi-squared test for categorical variables. p values were adjusted using the Hochberg procedure.

### Association of depressive symptoms with sarcopenia and obesity status

**Fig 1:**
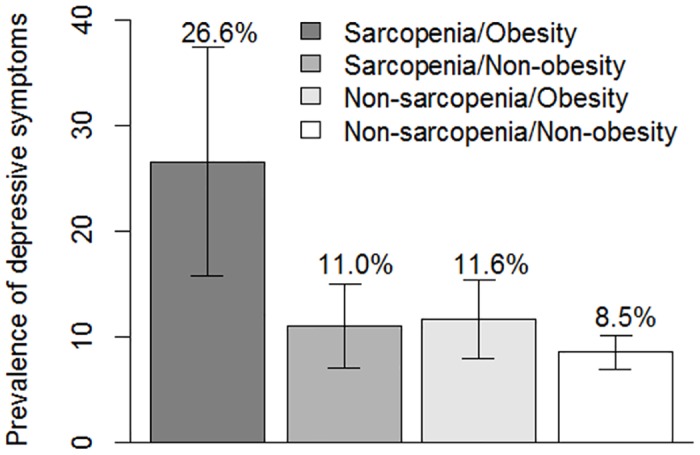
Prevalence of depressive symptoms with 95% confidence intervals by sarcopenia and obesity status in the Kashiwa study.

The prevalence of depressive symptoms in each of the four groups is shown in Fig 1. The prevalence of depressive symptoms was 26.6% in the sarcopenia/obesity group, which was the highest among the four groups.

In multiple logistic regression adjusted for age and sex, only the sarcopenia/obesity group had increased risk of depressive symptoms compared with the non-sarcopenia/non-obesity group, while neither the non-sarcopenia/obesity group nor the sarcopenia/non-obesity group had increased risk of depressive symptoms (Table 2, model 1). The significant association between sarcopenia/obesity and depressive symptoms (and the lack of significant association of depressive symptoms with sarcopenia/non-obesity or non-sarcopenia/obesity) persisted after successively adjusting for behavior variables (Table 2, model 2), social factors (Table 2, model 3), and medical variables (Table 2, model 4). In covariate-adjusted multiple logistic regression analysis with sarcopenia and obesity as two separate independent variables, the interaction term between sarcopenia and obesity was statistically significant (p = 0.04). In the age-stratified analysis, the presence of sarcopenia or obesity, or their combination was not associated with increased risk of depressive symptoms in participants aged 75 years or older (Table 2, model 4a). However, in participants aged 65 to 74 years, the sarcopenia/obesity group only had increased risk of depressive symptoms (Table 2, model 4b). Addition of BMI to these models did not attenuate the association between sarcopenia/obesity and depressive symptoms (data not shown).

**Table 2:** Adjusted odds ratio and 95% confidence intervals of depressive symptoms with sarcopenia and obesity status in the Kashiwa study.

	Sarcopenia				Non-sarcopenia		
	+ Obesity		- Obesity		+ Obesity		- Obesity^a^
	OR (95% CI)	p	OR (95% CI)	p	OR (95% CI)	P	OR
Model 1	3.63 (1.96–6.71)	<.001	1.23 (0.75–2.00)	0.41	1.39 (0.92–2.11)	0.12	-
Model 2	2.99 (1.58–5.65)	<.001	0.96 (0.58–1.59)	0.87	1.30 (0.85–1.97)	0.14	-
Model 3	3.03 (1.57–5.86)	0.001	0.96 (0.56–1.63)	0.87	1.34 (0.87–2.06)	0.19	-
Model 4	2.79 (1.43–5.43)	0.003	0.93 (0.55–1.60)	0.80	1.23 (0.79–1.91)	0.36	-
Model 4a	1.77 (0.75–4.18)	0.20	0.73 (0.35–1.54)	0.41	1.13 (0.55–2.33)	0.75	-
Model 4b	6.05 (1.89–19.38)	0.003	1.36 (0.63–2.93)	0.44	1.28 (0.72–2.26)	0.40	-

^a^ Non-sarcopenia/Non-obesity group is a reference group.

Abbreviations: OR, odds ratio; CI, confidence interval

Model 1: adjusted for age and sex

Model 2: adjusted for age, sex, food intake, poor sleep, and physical activity

Model 3: adjusted for age, sex, food intake, poor sleep, physical activity, education level, social isolation, living alone, and neighborhood ties

Model 4: adjusted for age, sex, food intake, poor sleep, physical activity, education level, social isolation, living alone, neighborhood ties, chronic comorbidity burden, use of antidepressant, and use of statin

Model 4a: adjusted for the same covariates as in Model 4, restricted to those aged 75 or over

Model 4b: adjusted for the same covariates as in Model 4, restricted to those aged 65 to 74

### Sensitivity analyses

**Fig 2:**
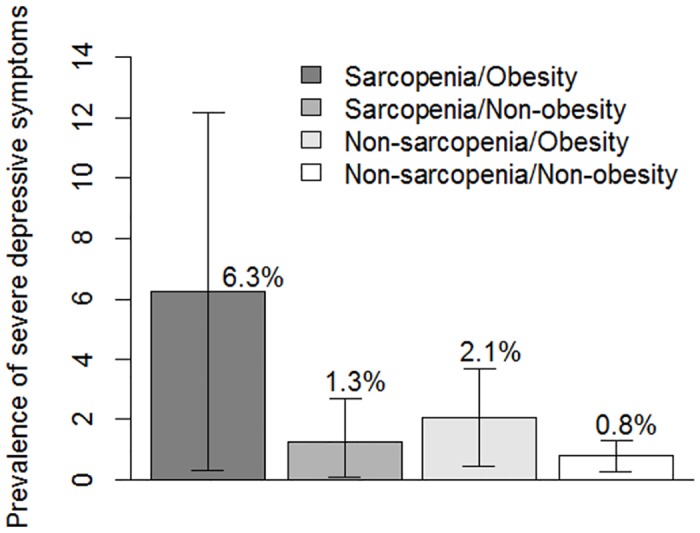
Prevalence of severe depressive symptoms with 95% confidence intervals by sarcopenia and obesity status in the Kashiwa study.

We repeated analyses with severe depressive symptoms as a dependent variable. The prevalence of severe depressive symptoms in each of the four groups is shown in Fig 2, and is much lower than that of depressive symptoms. Similarly, to the main analysis, only the sarcopenia/obesity group had increased risk of depressive symptoms compared with the non-sarcopenia/non-obesity group, while neither the non-sarcopenia/obesity group nor the sarcopenia/non-obesity group had increased risk of depressive symptoms in all the models, though the small number of participants with severe depressive symptoms resulted in a wider 95% CI (Table 3, model 1–4). The significant association between severe depressive symptoms and sarcopenia/obesity was also observed only in the age group between 65 and 74 years.

**Table 3:** Adjusted odds ratio and 95% confidence intervals of severe depressive symptoms with sarcopenia and obesity status in the Kashiwa study.

	Sarcopenia				Non-sarcopenia		
	+ Obesity		- Obesity		+ Obesity		- Obesity^a^
	OR (95% CI)	p	OR (95% CI)	p	OR (95% CI)	p	OR
Model 1	6.28 (1.75–22.48)	0.005	1.22 (0.30–4.94)	0.78	2.47 (0.87–7.04)	0.09	-
Model 2	4.55 (1.19–17.38)	0.03	1.03 (0.24–4.31)	0.97	2.02 (0.70–5.83)	0.20	-
Model 3	4.99 (1.25–19.90)	0.02	1.07 (0.24–4.69)	0.93	1.99 (0.67–5.85)	0.21	-
Model 4	4.16 (1.01–17.10)	0.04	1.05 (0.23–4.68)	0.95	1.71 (0.56–5.24)	0.35	-
Model 4a	2.08 (0.22–19.88)	0.52	0.89 (0.12–6.60)	0.91	1.48 (0.20–10.87)	0.70	-
Model 4b	20.21 (2.68–152.35)	0.004	-^b^	-	1.76 (0.41–7.62)	0.45	-

^a^ Non-sarcopenia/non-obesity group is a reference group.

^b^ No subject in the sarcopenia/non-obese group had severe depressive symptoms.

Abbreviations: OR, odds ratio; CI, confidence interval

Model 1: adjusted for age and sex

Model 2: adjusted for age, sex, food intake, poor sleep, and physical activity

Model 3: adjusted for age, sex, food intake, poor sleep, physical activity, education level, social isolation, living alone, and neighborhood ties

Model 4: adjusted for age, sex, food intake, poor sleep, physical activity, education level, social isolation, living alone, neighborhood ties, chronic comorbidity burden, use of antidepressant, and use of statin

Model 4a: adjusted for the same covariates as in Model 4, restricted to those aged 75 or over

Model 4b: adjusted for the same covariates as in Model 4, restricted to those aged 65 to 74

Additional analyses introducing four medical conditions (diabetes mellitus, stroke, heart disease, and cancer) as covariates did not attenuate the observed association between depressive symptoms and sarcopenia/obesity (data not shown).

Lastly, we conducted analysis excluding 67 participants with Mini-Mental State Exam score of 24 or lower (3.9%), which did not alter out conclusion (data not shown).

## Discussion

In this cross-sectional analysis of data from 1732 community-dwelling Japanese older men and women, we demonstrated that participants with sarcopenic obesity, the co-existence of sarcopenia and obesity, had significantly increased risk of depressive symptoms compared to those without either sarcopenia or obesity. This relationship remained statistically significant after successive addition of covariates to the model, albeit with slightly diminished effect size. Our findings suggest a synergistic impact on the risk of depressive symptoms exerted by sarcopenia and obesity in the elderly, since participants with either obesity or sarcopenia did not have increased risk of depressive symptoms and the interaction term between sarcopenia and obesity was statistically significant.

The mechanisms underlying the association between depressive symptoms and sarcopenic obesity remain elusive and largely unknown. However, we speculate that there are several biological mechanisms that could potentially mediate the depression-sarcopenic obesity association. Obesity may cause a chronic inflammatory state [30] and insulin resistance [31], which, in turn, are associated with both sarcopenia [32,33] and depression [34,35]. Elevated leptin has been observed in those with sarcopenia [36]. and was associated with increased risk of depression, especially in the presence of obesity [37]. Therefore, leptin resistance might be implicated as a mediating factor for the association between sarcopenic obesity and depression. There are other clinical factors that need to be considered. Obesity may lead to depression through its negative effects on self-image, disordered eating, and its associated stigma [38].

Several previous studies have shown the association of depression with physical performance [39] such as gait speed [13,14] or hand grip strength [15], consistent with our findings. With regard to the association between obesity and depression, previous findings are inconsistent (3–6), with some studies demonstrating positive association, compatible with our findings (3). Most previous studies used BMI to ascertain obesity and our findings should be corroborated in future studies using percentage body fat to evaluate body adiposity.

We demonstrated that the association between sarcopenic obesity and depressive symptoms varies depending on age. The heightened risk of depressive symptoms associated with sarcopenic obesity was mostly observed in participants aged between 65 and 74 years, and it was not statistically significant in participants aged 75 years or older. Several recent studies have shown that the effects of cardiovascular risk factors may have different impacts in the elderly or frail population when compared to younger people. Obesity, or higher BMI, was inversely associated with mortality in elderly hospitalized patients [7]. Elevated blood pressure was associated with lower mortality risk in physically frail elderly adults who could not walk 20 feet [27]. Metabolic syndrome was associated with a lower probability of prevalent and incident functional disability in older adults [28]. The association between metabolic syndrome and cardiovascular events was observed only in patients younger than 75, but not in patients aged 75 or over [29]. Our study findings are consistent with these studies in that the adverse effects of obesity, one of the cardiovascular risk factors, is limited to relatively younger participants, but extend these studies by showing that the age difference in the adverse effects of obesity is also observed in a mental aspect. The reasons for the lack of impact of sarcopenic obesity on depressive symptoms among participants aged 75 years or older were unclear and need to be investigated in future studies. Those with depressive symptoms, particularly at extreme age, would be less likely to participate in epidemiological studies, and this possibility of healthy participant bias should be explored.

Our study has several limitations. First, we employed GDS to ascertain depressive symptoms. Although GDS is a valid measure of depressive symptoms and is widely used in clinical practice, it was originally developed as a screening tool rather than a diagnostic tool. It is possible that the depressive symptoms identified with GDS could be related to physical illness and not depression. However, the GDS has been successfully used for older adults in various settings and shown to have satisfactory psychometric properties [40]. In addition, the relationships between depressive symptoms and sarcopenic obesity were replicated when severe depressive symptoms, defined by a higher cutoff point of the GDS, was employed as an outcome. The higher cutoff point indicates more severe depression symptomatology and may increase the specificity (i.e., those identified as having severe depressive symptoms were most likely to be truly depressed). The successful replication with a more specific definition of depressive symptoms may support the validity of our findings. Second, our study was a cross-sectional study, and the observed associations could be confounded by unmeasured or uncontrolled variables. In addition, data in the present study did not provide any explanation for the underlying mechanisms of the observed associations or the direction of the causality. Future studies with a prospective longitudinal cohort design will be needed to establish a causal relationship between depressive symptoms and sarcopenic obesity. Finally, the participants were exclusively functionally-independent, community dwelling Japanese older adults. The generalizability of our study findings will need to be tested using data from participants with a greater comorbidity burden or from other racial/ethnic groups.

In conclusion, we demonstrated that depressive symptoms was positively associated with sarcopenic obesity in functionally independent Japanese older adults. Sarcopenia and obesity appear to exert a synergistic impact on the risk of depressive symptoms, since either sarcopenia or obesity alone was not associated with depressive symptoms. Future research will need to be conducted to establish the causal pathways and identify mediators of the association, with particular attention to modifiable factors, so that co-occurrence of these two disorders, depression and sarcopenic obesity, could be prevented.
